# (E)-2-methoxy-4-(3-(4-methoxyphenyl) prop-1-en-1-yl) Phenol Ameliorates MPTP-Induced Dopaminergic Neurodegeneration by Inhibiting the STAT3 Pathway

**DOI:** 10.3390/ijms20112632

**Published:** 2019-05-29

**Authors:** Ji Yeon Choi, Jaesuk Yun, Chul Ju Hwang, Hee Pom Lee, Hae Deun Kim, Hyungok Chun, Pil-Hoon Park, Dong Young Choi, Sang-Bae Han, Jin Tae Hong

**Affiliations:** 1 College of Pharmacy and Medical Research Center, Chungbuk National University, 194-31 Osongsaemgmyeong 1-ro, Cheongju 28160, Korea; cjy8316@hanmail.net (J.Y.C.); hcj0629@naver.com (C.J.H.); heepom@empal.com (H.P.L.); khdsun62@naver.com (H.D.K.); hyungok@korea.kr (H.C.); shan@chungbuk.ac.kr (S.-B.H.); 2 College of Pharmacy, Yeungnam University, 280, Daehak-ro, Gyeongsan, Gyeongbuk 38541, Korea; parkp@yu.ac.kr (P.-H.P.); dychoi@yu.ac.kr (D.Y.C.)

**Keywords:** Parkinson’s disease, neuroinflammation, MMPP, mitogen-activated protein kinase, signal transducer and activator of transcription 3

## Abstract

Neuroinflammation is implicated in dopaminergic neurodegeneration. We have previously demonstrated that (E)-2-methoxy-4-(3-(4-methoxyphenyl) prop-1-en-1-yl) phenol (MMPP), a selective signal transducer and activator of transcription 3 (STAT3) inhibitor, has anti-inflammatory properties in several inflammatory disease models. We investigated whether MMPP could protect against 1-methyl-4-phenyl-1,2,3,6-tetrahydropyridine (MPTP)-induced dopaminergic cell loss and behavioral impairment. Imprinting control region (ICR) mice (8 weeks old, n = 10 per group) were administered MMPP (5 mg/kg) in drinking water for 1 month, and injected with MPTP (15 mg/kg, four times with 2 h intervals) during the last 7 days of treatment. MMPP decreased MPTP-induced behavioral impairments in rotarod, pole, and gait tests. We also showed that MMPP ameliorated dopamine depletion in the striatum and inflammatory marker elevation in primary cultured neurons by high-performance liquid chromatography and immunohistochemical analysis. Increased activation of STAT3, p38, and monoamine oxidase B (MAO-B) were observed in the substantia nigra and striatum after MPTP injection, effects that were attenuated by MMPP treatment. Furthermore, MMPP inhibited STAT3 activity and expression of neuroinflammatory proteins, including ionized calcium binding adaptor molecule 1 (Iba1), inducible nitric oxide synthase (iNOS), and glial fibrillary acidic protein (GFAP) in 1-methyl-4-phenylpyridinium (MPP^+^; 0.5 mM)-treated primary cultured cells. However, mitogen-activated protein kinase (MAPK) inhibitors augmented the activity of MMPP. Collectively, our results suggest that MMPP may be an anti-inflammatory agent that attenuates dopaminergic neurodegeneration and neuroinflammation through MAO-B and MAPK pathway-dependent inhibition of STAT3 activation.

## 1. Introduction

Parkinson’s Disease (PD) is a major age-related progressive movement disorder. The prevalence of PD increases with age, affecting 1.6% of the population over the age of 65 years old [1]. Its major symptoms are slow physical movements, muscle rigidity, and postural instability. Cognitive dysfunction is also a common symptom of PD. PD is characterized by the loss of dopamine neurons in the substantia nigra, with decreased striatal dopamine levels and consequent extrapyramidal motor dysfunction [2,3]. For study of PD, 1-methyl-4-phenyl-1,2,3,6-tetrahydropyridine (MPTP)-injected animal models are used widely. MPTP has been shown to induce dopamine (DA) neuron degeneration. Mice treated with MPTP share specific biological features with those of PD, including dopamine depletion in the striatum and loss of dopaminergic neurons in the substantia nigra [4,5]. MPTP can cross the blood–brain barrier and is converted to its toxic metabolite, the 1-methyl-4-phenylpyridinium (MPP^+^) ion, by monoamine oxidase B (MAO-B) [5,6]. Accumulation of MPP^+^ in mitochondria results in oxidative damage of neurons, causing apoptotic or necrotic cell death [2,7,8,9,10]. MPTP-induced dopaminergic neurotoxicity was linked to rapid activation of signal transducer and activator of transcription 3 (STAT3) in striatal astrocytes [11,12]. STAT3 is implicated in brain development and pro-inflammatory responses in microglia [13]. STAT3 is a critical transcription factor for the regulation of monoamine oxidase A [14,15]. Yuan et al. reported that microglia-derived inflammation and behavioral changes in PD models can be suppressed via inhibiting the mitogen-activated protein kinase (MAPK) and STAT3 pathways [16]. The MAPK family regulates various cellular activities such as proliferation, differentiation, survival, and apoptosis [17]. The MAPK pathway in neurons is implicated in neurodegenerative diseases like Alzheimer’s disease (AD), PD, and amyotrophic lateral sclerosis (ALS) [18,19,20]. JNK and p38 play essential roles in apoptosis of dopaminergic neurons [21]. It was observed that striatal dopaminergic nerve terminals in MPTP-treated JNK-deficient mice were more resistant than those in MPTP-treated wild type mice [22]. Furthermore, MAPK signaling pathways are important for MAO-B gene expression, since the dominant negative JNK and p38 can selectively inhibit MAO-B promoter activation [23]. 

Inactivation of the MAPK and STAT3 pathways using therapeutic agents such as nitidine, ginkgetin [24], and dexmedetomidine [25] may be therapeutically useful for PD. We have demonstrated that (E)-2,4-bis(p-hydroxyphenyl)-2-butenal (BHPB) possesses anti-inflammatory properties by inhibiting STAT3 activation in arthritis [26], cancer [27], and AD [28]. The upgraded BHPB analogue, (E)-2-methoxy-4-(3-(4-methoxyphenyl) prop-1-en-1-yl) phenol (MMPP) [29], binds strongly to STAT3 through a direct molecular interaction with the hydroxyl residue in the core fragment of STAT3, thereby inhibiting activation of STAT3. Here, we investigated whether MMPP could prevent MPTP-induced neurodegenerative damage by inhibiting MAPK/STAT3 activation.

## 2. Results

### 2.1. Effect of MMPP in MPTP-Induced Behavioral Impairments

**Figure 1:**
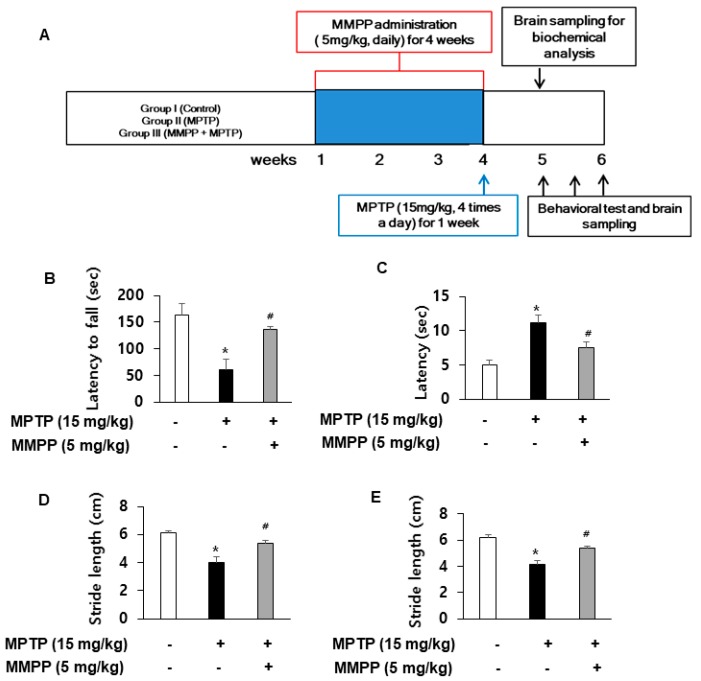
(E)-2-methoxy-4-(3-(4-methoxyphenyl) prop-1-en-1-yl) phenol (MMPP) ameliorates 1-methyl-4-phenyl-1,2,3,6-tetrahydropyridine (MPTP)-induced behavior disorder: Performance on the rotarod is impaired in MPTP-injected groups. The drug administration and experimental schedule is shown (**A**). The impairment is ameliorated in MPTP-injected MMPP-treated groups (**B**). MPTP-induced bradykinesia is ameliorated in MPTP-injected MMPP treated groups (**C**). Stride lengths of forelimbs (**D**) and hindlimbs (**E**) are increased by MMPP treatment in MPTP-injected groups. Values are presented as mean ± SD from 10 mice. *p < 0.05, significant difference from saline-injected mice; # *p* < 0.05, significant difference between MPTP-injected groups.

Motor coordination and balance capability were evaluated with the rotarod test. MPTP treatment reduced the latency to fall from a treadmill compared with that of the control group. However, the decrement in latency was significantly rescued in MMPP-pretreated/MPTP-treated mice (137.0 ± 4.36) compared to MPTP-treated mice (60.75 ± 20.36) (Figure 1B). To examine the motor deficits induced by MPTP administration, we conducted a pole test and measured the time to reach the base of the pole. The time to descend was significantly delayed by MPTP injection. However, the delay was significantly less in MMPP-pretreated/MPTP-treated mice (7.50 ± 0.87) compared to MPTP-treated mice (11.25 ± 1.11) (Figure 1C). A reduction in stride length was observed in both forelimbs (Figure 1D) and hindlimbs of the MPTP-injected group (Figure 1E). The stride length was longer in MMPP-pretreated/MPTP-treated mice (forelimb: 5.37 ± 0.19; hindlimb: 5.39 ± 0.11) compared to MPTP-treated mice (forelimb: 4.03 ± 0.36; hindlimb: 4.17 ± 0.29).

### 2.2. Effect of MMPP on the Expression of Astrogliosis and Microgliosis-Related Proteins

**Figure 2:**
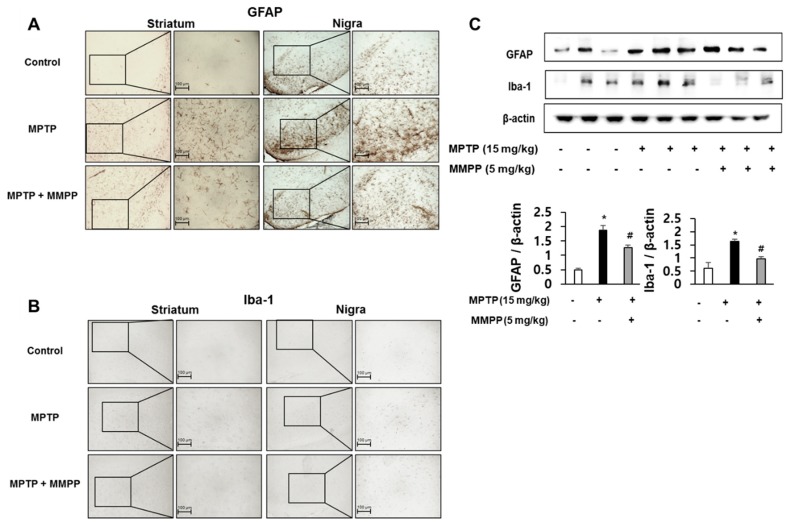
MMPP reduces glial fibrillary acidic protein (GFAP) and Iba-1 expression. The effects of MMPP on reactive astrocytes and reactive microglial cells were measured by immunohistochemical analysis. Immunostaining for GFAP in the striatum and substantia nigra in 20 μm-thick sections of mouse brains with specific primary antibodies and biotinylated secondary antibodies (**A**). Immunostaining of Iba-1 in the striatum and substantia nigra in 20 μm-thick sections of mouse brains with specific primary antibodies and biotinylated secondary antibodies (**B**). The representative stained tissues were viewed with a microscope (50× or 200× magnification). (**C**) Expressions of GFAP and Iba-1 in the substantia nigra were measured by immunoblotting. Values are presented as mean ± SD from 10 mice. *p < 0.05, significant difference from saline-injected mice; #*p* < 0.05, significant difference between MPTP-injected groups.

Immunohistochemistry and western blotting were performed to evaluate whether MPTP injection could induce neuroinflammation and activate astrocytes and/or microglia. GFAP (a marker of astrocytic activation) and Iba-1 (a marker of microglial activation) expression were examined in mouse brains. GFAP-positive (Figure 2A) and Iba-1-positive (Figure 2B) cell numbers in the striata and substantia nigra of MMPP-pretreated/MPTP-treated mice were significantly lower than those in MPTP-treated mice. The MPTP-induced elevations in expression of GFAP and Iba-1 in the substantia nigra were significantly decreased in MMPP-pretreated/MPTP-treated mice compared to MPTP-injected mice (Figure 2C).

### 2.3. Effect of MMPP on Dopaminergic Neurodegeneration

**Figure 3:**
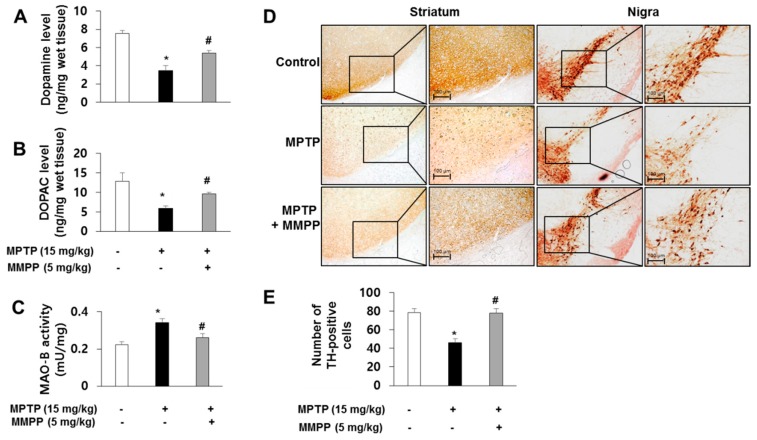
MMPP ameliorates MPTP-induced dopaminergic neurodegeneration. The levels of dopamine (**A**) and 3,4-Dihydroxyphenylacetic acid (DOPAC) (**B**) in mouse brains were determined by HPLC. Monoamine oxidase B (MAO-B) activity was determined with an assay kit (**C**). The effect of MMPP on tyrosine hydroxylase (TH)-positive neurons was measured by immunohistochemistry (**D** and **E**). Representative stained tissues were viewed with a microscope (50× or 200× magnification). Values are presented as mean ± SD from 10 mice. *p < 0.05, significant difference from saline-injected mice; #*p* < 0.05, significant difference between MPTP-injected groups.

**Figure 4:**
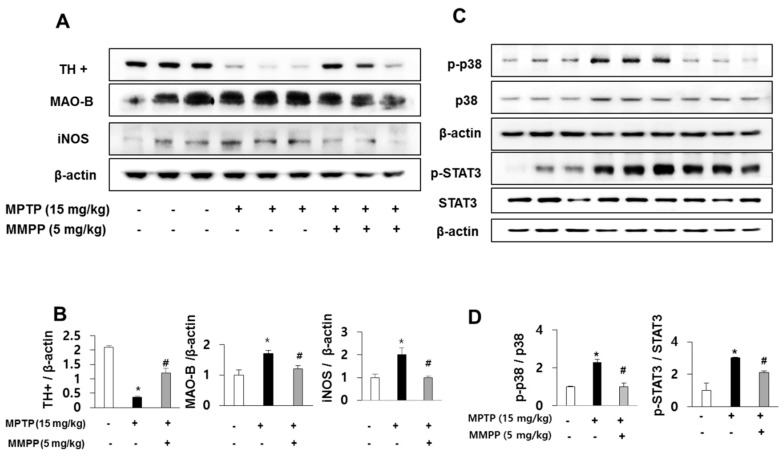
Effect of MMPP on MPTP-induced protein expressions. The effects of MMPP on dopaminergic neuronal cell marker and inflammatory marker proteins were measured by western blotting. Expressions of TH+, MAO-B, and iNOS in the substantia nigra were measured (**A** and **B**). Expressions of p-p38 and p-STAT3 in the substantia nigra were measured (**C** and **D**). Values are presented as mean ± SD from 10 mice. * *p* < 0.05, significant difference from saline-injected mice; # *p* < 0.05, significant difference between MPTP-injected groups.

To investigate whether MMPP-induced inactivation of astrocytes could prevent dopamine depletion, we examined the level of DA and its metabolites. To measure the levels of dopamine and DOPAC, quantification was performed with HPLC analysis of striatal tissue. DA and DOPAC levels in the striata of MPTP-injected MMPP-treated mice were significantly increased compared to those in MPTP-treated mice (Figure 3A,B). We also examined MAO-B activity and observed that elevated MAO-B activity by MPTP was reduced by MMPP in the substantia nigra (Figure 3C). We evaluated MPTP neurotoxicity using immunohistochemistry and western blotting for TH. MPTP-induced protein expression of TH in brain tissue was significantly increased in MMPP-treated mice compared to MPTP-injected mice (Figure 4A,B). Decreased numbers of TH-positive cells were recovered from the brains of MMPP-pretreated/MPTP-treated mice compared to MPTP-injected mice (Figure 3D,E). 

### 2.4. Effect of MMPP on MAO-B Expression and Activation of STAT3

**Figure 5:**
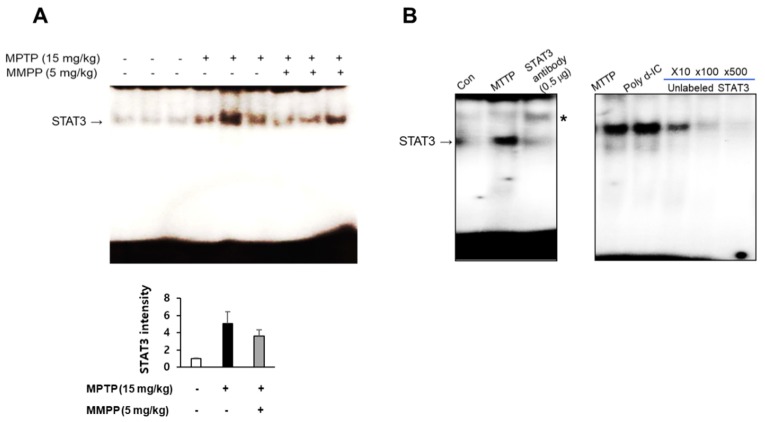
MMPP reduces translocation of STAT3. The protective effects of MMPP against inflammatory signaling pathways were measured by EMSA. MMPP inhibited MPTP-induced translocation of STAT3 protein to the nucleus in brain tissue, as determined (**A**). Values are presented as mean ± SD from three mice. Specificity of STAT3 DNA binding activity. * indicates super shift of STAT3 (**B**, left panel). Noncompetitive (Poly d-IC) and competitive (with unlabeled oligonucleotide of STAT3) assays (**B**, right panel).

As MAO B-is responsible for the conversion of MPTP to MPP^+^, we examined MAO-B expression using western blot. MPTP-induced expression of MAO-B was significantly lower in the brains of MMPP-pretreated/MPTP-treated mice than those of MPTP-treated mice (Figure 4A,B). As MAO-B expression in astrocytes is responsible for MPTP metabolism and is induced by MAPK signaling, which is accompanied by reactive astrogliosis, we conjectured that downregulated expression of MAO-B in MMPP-pretreated/MPTP-treated mice may be related to inactivation of the p38 MAPK pathway. Furthermore, MPTP-induced phosphorylation of p38 was significantly reduced in MMPP-treated mouse brains compared to those of MPTP-treated mice (Figure 4C,D). To determine whether MMPP treatment reduced neuroinflammation, we examined iNOS expression using western blotting. MPTP-induced expression of iNOS was significantly lower in the brains of MMPP-pretreated/MPTP-treated mice (Figure 4A,B). To investigate whether MMPP treatment could inhibit the activation of STAT3 after MPTP injection, we measured STAT3 expression by western blotting, and DNA binding activity by EMSA. MPTP-induced expression of p-STAT3 was significantly lower in the brains of MMPP-pretreated/MPTP-treated mice (Figure 4C,D). Translocation of STAT3 was also inhibited in the brains of MMPP-treated mice than in MPTP-treated mice (Figure 5A). STAT3 signaling is triggered by translocation of STAT3 to the nucleus. Upregulated levels of STAT3 in the nucleus fraction were induced by MPTP. However, MMPP reduced STAT3 levels. These results indicate that STAT3 signaling is inhibited by MMPP. The specificity of STAT3 DNA binding activity was confirmed with the super shift assay and competitive and noncompetitive assays (Figure 5B). The STAT3 antibody may create a complex with STAT3 and induce slower migration, which inhibits the DNA binding activity of STAT3. Furthermore, STAT3 translocation was observed in noncompetitive (Poly d-IC) assays, but not in competitive (with dose-dependence of unlabeled oligonucleotide of STAT3) assays. These results showed the specificity of the DNA binding activity of STAT3 in this study. 

### 2.5. Effect of MMPP on MPP^+^-Induced Inflammation in Primary Cultured Cells

**Figure 6:**
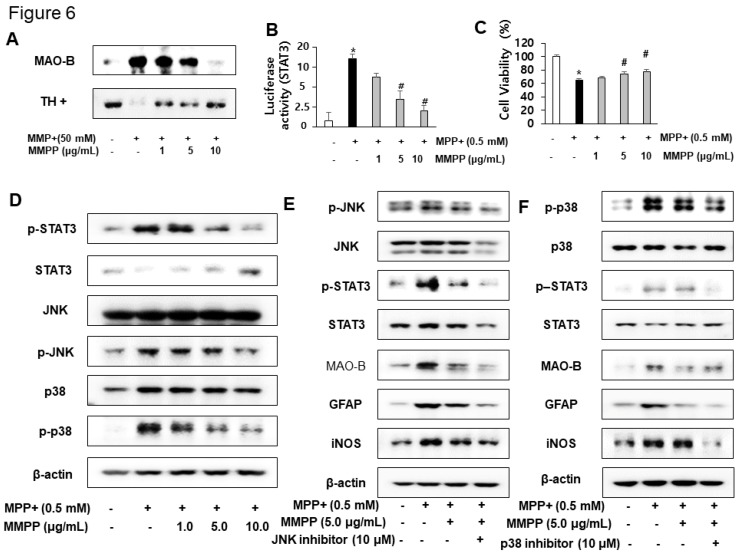
Effect of MMPP against dopaminergic neurodegeneration in vitro. The protective effects of MMPP against neurodegeneration, and expressions of TH, MAO-B, and inflammatory marker proteins were measured by western blotting analysis (**A**, **D**, **E**, and **F**). STAT3 luciferase activity was determined as described in the Methods (**B**). To evaluate the protective effects of MMPP, cell viability was measured using the LDH assay kit (**C**). Activation of STAT3, p38, and JNK were detected by western blotting (**D**). To evaluate the involvement of JNK (**E**) and p-38 (**F**) pathways in the inhibitory effect of MMPP on 1-methyl-4-phenylpyridinium (MPP^+^)-induced neuroinflammation, cultured primary cells were incubated with specific JNK and p-38 inhibitors (10 μM) for 24 h and co-treated with MMPP (5 μg/mL) at 37 °C. These effects were investigated by western blot analysis. Expression levels of MAO-B, STAT3, and inflammatory markers (GFAP and iNOS) were measured (**E** and **F**). *p < 0.05, significant difference from control astrocytes; #*p* < 0.05, significant difference from the MPP^+^-treated astrocytes.

Although MMPP increased TH expression, MMPP treatment decreased MPP^+^-induced MAO-B expression (Figure 6A). To evaluate the effects of MMPP on MPP^+^-induced inflammation, we first investigated STAT3 luciferase activity. We observed that MMPP attenuated MPP^+^-induced STAT3 transcriptional activity (Figure 6B). As MPP^+^ promotes cell apoptosis, we investigated the preventive effect of MMPP. MPP^+^-induced reduction of cell viability (63 ± 4.3%) was recovered by MMPP treatment in a concentration-dependent manner in primary cultured cells (Figure 6C). Moreover, MPP^+^-induced expression of p-STAT3, p-JNK, and p-p38 were dose-dependently decreased by MMPP treatment in cultured cells (Figure 6D). To study the involvement of MAPK signaling, we employed specific inhibitors of JNK and p38 in the presence of MMPP (5 μg/mL). MMPP decreased MAO-B and pro-inflammatory protein (iNOS and GFAP) expression following MPP^+^ treatment in cultured cells. The MMPP-induced inhibitory effect was amplified by co-treatment with p38 and JNK inhibitors in cultured cells (Figure 6E,F).

## 3. Discussion

Epidemiological studies and experimental evidence have indicated that neuroinflammation and oxidative stress may trigger the initial occurrence and promote progression of PD [30]. Activation of microglia and astrocytes observed in PD patients and PD animal models suggests that neuroinflammation plays an important role in the progression of PD [31]. Therefore, anti-inflammatory agents may be therapeutically beneficial for PD. For instance, naloxone [32] inhibits microglial activation and reduces superoxide production in the lipopolysaccharide-induced PD model, and vasoactive intestinal peptide [33] blocks microglial activation by decreasing THF-α, IL-1β, and iNOS expression in the MPTP-induced PD model. In our previous study, an anti-inflammatory agent, thiacremonone, suppressed PD progression in the MPTP-induced PD model [34]. In addition, astrogliosis is strongly associated with neuroinflammation in PD [35]. We demonstrated that MMPP rescued behavioral deficits in motor coordination, loss of DA- and TH-positive neurons, astrocyte activation, and neuroinflammation-induced by MPTP. In an in vitro study, MMPP effectively decreased MPP^+^-induced inflammatory mediator generation in primary cultured cells, and dose-dependently attenuated cell death. These results suggest that MMPP has an anti-inflammatory activity and reduces neuroinflammation-induced by MPTP. 

Next, we studied a mechanism underlying the anti-inflammatory activity of MMPP. MPTP is converted to MPP^+^ by MAO-B in astrocytes, which could cause neuronal cell death with increased levels of MPP^+^ [36]. Accumulated evidence suggest that the inhibition of MAO-B may protect against MPTP-generated MPP^+^ toxicity [37,38,39]. MAO-B inhibitors such as selegiline [40] and rasagiline [41] attenuate MPP^+^-induced toxicity by blocking the metabolism of DA, thereby improving PD motor symptoms. The MAO-B inhibitors have the potential to promote neuroprotection in PD. In the present study, MMPP significantly inhibited MPTP-induced decreases of TH expression and increases of MAO-B expression and activation in vivo. These data suggest that MMPP could prevent dopaminergic neurodegeneration by MPTP through inhibition of MAO-B.

Recent studies have reported that activation of the p38 and JNK signaling pathways plays an important role in neurodegeneration in MPTP-induced PD animal and PD patient brains [20,42]. JNK3-null mice have decreased MPTP-induced damage in dopaminergic neurons, suggesting that the JNK signaling pathway may play a major physiological role in neuronal function [22]. Moreover, the p38, JNK, and MAPK signaling pathways are associated with MAO-B gene expression [43,44]. MAO-B expression was significantly lower in MMPP-treated mouse brains than in MPTP-treated mouse brains, an effect mediated by inactivation of the p38 pathway. However, in in vitro studies, we observed that MMPP also has a protective role in neuronal culture. In this culture system, MPP^+^ treatment increased MAO-B and GFAP expression levels. GFAP is a marker of astrocytes, and this increase is similar to a previous report [45]. Therefore, MMPP may inhibit MAO-B associated p38, JNK, and MAPK signaling pathways directly. 

Additionally, the combined treatment of MMPP and a p38-specific inhibitor (10 μM) decreased the activation of astrocytes compared to that in MMPP-only treated cells. MMPP decreased MAO-B expression in cultured cells by downregulating the activation of the p38 and JNK pathways. The transcriptional activity of STATs requires serine phosphorylation mediated by serine or threonine kinases of other signaling pathways, including MAPK. Activated MAPKs can phosphorylate nuclear transcription factors, causing regulation of metabolism [46]. Thus, increased inhibition of p38 and JNK pathways can block STAT3 serine phosphorylation. In our study, anti-neuroinflammatory effects were amplified when p38 and JNK inhibitors were co-administered with MMPP. Furthermore, the p38 and JNK inhibitors downregulated the protein expression level of phosphorylated STAT3. We demonstrated that STAT3 activation was inhibited by MMPP and inhibitors of p38 and JNK. Further, p38 and JNK regulated the STAT3 phosphorylation in a dose-dependent manner.

Both in vitro and in vivo serine phosphorylation of STAT3 were observed, together with constitutive activation of p38 and JNK. The STAT3 pathway contributes to inflammation-mediated astrogliosis with activated microglia. Agents inhibiting STAT3 phosphorylation may therefore be promising candidate drugs. For instance, nitidine significantly suppresses microglial activation via the STAT3 pathway and improves behavioral function in PD animal models [16]. Triptolide downregulates the activation of astrocytes by suppressing inflammation in the substantia nigra in a rat model of PD by blocking the STAT3 pathway [47]. Furthermore, inhibition of the p38 and JNK pathways suppresses the transcriptional activity of STAT3 [46]. p38 is a key regulator of STAT3 phosphorylation in macrophages [48]. In our study, MMPP inhibited STAT3 DNA binding and transcriptional activity, suggesting that MMPP can inhibit neuroinflammation through inactivation of astrocytes via blocking STAT3-mediated p38 and JNK MAPK pathways. Previously, we reported that MMPP has anti-inflammatory effects in inflammatory disease models such as AD, rheumatoid arthritis, and cancer [29,49,50]. We did not detect any side effects of MMPP with the effective dose of MMPP (5 mg/kg) during treatment for 30 days [29]. Moreover, MMPP has a good rule of five—chemistry, manufacturing, and control (CMC)-like rule, World Drug Index (WDI)-like rule, Caco2 cell permeability, and plasma protein binding [29]. Further, MMPP has good aqueous solubility, human intestinal absorption, and skin permeability [29]. These findings indicate that MMPP may be beneficial for the treatment of inflammatory diseases with low toxicity. We propose that MMPP could be a promising agent for the prevention of PD.

## 4. Materials and Methods

### 4.1. Animals

Male imprinting control region (ICR) mice (RRID: IMSR_TAC:icr, 8 to 10 weeks old, Hajinbiotech, Gyeonggi-do, Korea) were maintained and handled. The experimental protocols were carried out according to the guidelines for animal experiments of the Institutional Animal Care and Use Committee (IACUC) of the Laboratory Animal Research Center at Chungbuk National University, Korea (CBNUA-1073-17-01, 01MAR2017). All efforts were made to minimize animal suffering and to reduce the number of animals used. All mice were housed in a room with automatic temperature control (21–25 °C) and relative humidity (45–65%), with a 12 h light/dark cycle. Food and water were available ad libitum. All studies were approved by and performed according to the ethical guidelines of the Chungbuk National University Animal Care Committee.

### 4.2. Materials

To synthesize MMPP, 4-Iodo-2-methoxyphenol (500 mg, 2 mmol, Sigma Aldrich, St. Louis, MO, USA) and 4-allylanisole (296.4 mg, 2 mmol, Sigma Aldrich) were added to triphenylphosphine (105 mg, 0.4 mmol), Pd(OAc)_2_ (44.9 mg, 0.2 mmol), and tributylamine (451 μL, 1.9 mmol) in a 25 mL round bottom flask. The reaction mixture was stirred for 2 h at 45 °C in an argon atmosphere. The product was purified by flash silica gel chromatography using hexane and ethyl acetate (3:1 mixture *v*/*v*) as the mobile phase. Reduction of the alkene or aldehyde of α, β-unsaturated aldehyde moieties resulted in stable compounds to protect the phenolic alcohol from ether. The purity of MMPP was over 99.5%, as determined with HPLC analysis.

### 4.3. MPTP Injections

Computer-generated random numbers were used for simple randomization of animals. There were three groups (n = 10 per group): (I) Control group, (II) MPTP group, and (III) MMPP + MPTP group. MMPP was given to group (III) in drinking water daily at a dose of 5 mg/kg for 4 weeks. Mice received an intraperitoneal injection of MPTP (15 mg/kg, TOCRIS) or saline four times a day for 7 days with 2 h intervals. We performed behavioral tests 7 days after the MPTP injections to examine whether there were differences in neurotoxin-induced behavioral deficits between MPTP-treated mice and MMPP-treated mice. Rotarod, pole, and gait tests were conducted as described below. A graphical timeline of the experiment is shown in Figure 1A. MPTP and MMPP were prepared by blinded experimenters.

### 4.4. Rotarod Test

We performed behavioral tests 2 days after the last MPTP injection to examine whether there were differences in neurotoxin-induced behavioral deficits between the MPTP-treated group and control group. The rotarod test was performed as described previously using the rotarod treadmill (MED Associates Inc., St. Albans, VT) [34]. Mice were trained for two consecutive days before MPTP injections in acceleration mode (2–20 rpm) over 5 min. The training was repeated with a fixed speed (16 rpm) until the mice were able to stay on the rod for at least 60 s. 

### 4.5. Pole Test

The pole test was performed as described previously using a rough-surfaced wooden pole (1 cm in diameter, 55 cm in height) [34]. The test trials were performed three times per animal, and average values from three examinations were used for each animal. Briefly, a rough-surfaced wooden pole (1 cm in diameter, 55 cm in height) was placed vertically on the floor of the home cage. Upon being placed head-upward on top of the pole, mice turned downward and descended back to their home cages. After three repeats of the training trial, the test trials were performed. During the test trials, the total time for them to orient downward and descend to the floor was assessed. 

### 4.6. Gait Test

The gait test was performed as described previously using a bright and dark runway box [34]. Mice were acclimatized to the new environment by two training trials. A single test trial was performed, and stride length was measured as the distance between successive paw prints. Data are presented as the average of five strides for each animal.

### 4.7. Immunohistochemical Staining

After being transferred to 30% sucrose solution, brains were cut into 20 μm sections using a cryostat microtome (Leica CM 1850; Leica Microsystems, Seoul, Korea). After two washes in phosphate-buffered saline (PBS) (pH 7.4) for 10 min each, and endogenous peroxidase activity was quenched by incubating the samples in 3% hydrogen peroxide in phosphate-buffered saline (PBS) for 20 min, followed by two washes in PBS for 10 min each. The brain sections were blocked for 1 h in 5% bovine serum albumin (BSA) solution and incubated overnight at 4 °C in primary antibody against tyrosine hydroxylase (TH) (AB1542, RRID:AB_90755, 1:500, 2 μg/mL, Millipore, Burlington, MA, USA), glial fibrillary acidic protein (sc-33673, RRID:AB_627673, 1:200, 1 μg/mL, Santa Cruz Biotechnology Inc., Santa Cruz, CA, USA) or Iba-1 (019-19741, RRID:AB_839504, 1:300, 2 μg/mL, Wako Co., Nagano, Japan) at room temperature for 2 h. After multiple washes with PBS, the sections were incubated for 1 h in appropriate biotinylated secondary antibodies (1:1000, Vector Laboratories, Burlingame, CA, USA). Brain sections were washed three times in PBS for 10 min each, and visualized by a chromogenic 3,3’-diaminobenzidine (DAB, Vector Laboratories) reaction for up to 10 min. Sections were dehydrated in a series of graded alcohols, cleared in xylene, and coverslipped using Permount. 

The total number of TH-positive cells was counted in sections according to previous reports [34]. Briefly, every sixth section throughout the entire extent of the substantia nigra was picked, and immunostaining for TH was performed. The number of TH-positive neurons was counted by using a computer-assisted image analysis system consisting of a Zeiss Axioskop2 Plus photomicroscope equipped with an MS-2000 (Applied Scientific Instrumentation, Eugene, OR, USA) computer-controlled motorized stage, a Sony DXC-390 video camera, a DELL GX260 workstation, and the BIOQUANT Stereology Toolkit Plug-in for BIOQUANT Nova Prime software (BIOQUANT Image Analysis Corporation, Nashville, TN, USA). The substantia nigra region was observed at a low magnification (10× objective), and was outlined by using a set of anatomical landmarks. The cell number was counted at a high magnification (50× objective). Representative images (200× objective) are also shown.

### 4.8. HPLC Analysis of Dopamine and DOPAC

Dopamine and 3,4-Dihydroxyphenylacetic acid (DOPAC) in the striatum were measured as described previously [51] by a two-channel electrochemical detector (Waters Associates). After centrifugation (15,000× *g*, 30 min, 4 °C), the supernatant was diluted with the mobile phase (75 mM of NaH_2_PO_4_, 1.7 mM octane sulfonic acid, and 10% methanol; pH 3.0), and 10 μL of the sample was isocratically eluted through an 80 × 4.6 mm C18 column with flow rate of 1.5 mL/min. Concentrations were normalized by wet tissue weight.

### 4.9. Nuclear Extraction and Gel Mobility Shift Assay

Gel mobility shift assay was conducted using a slight modification of a previously described method [52]. In brief, 10 μg of nuclear protein of brain tissue was incubated in 25 μL of the total volume of incubation buffer (10 mmol/L Tris; pH 7.5; 100 mmol/L NaCl, 1 mmol/L dithiothreitol, 4% glycerol, 80 mg/L salmon sperm DNA) at 4 °C for 15 min, followed by another 20 min incubation with 9.25 mBq [-^32^P] of an ATP-labeled oligonucleotide containing the STAT3 binding site at room temperature. The sense oligonucleotide sequence was 5-AGAAACAGGATGGCCCAATGG-3. The DNA–protein binding complex was electrophoretically resolved on a 6% nondenatured polyacrylamide gel at 150 volts for 90 min. The gels were dried and autoradiographed using Kodak MR film at −80 °C overnight.

### 4.10. Primary Cell Culture

Sprague–Dawley rats (Hajinbiotech, Gyeonggi-do, Korea) were maintained in accordance with the guidelines for animal experiments of IACUC. Sprague–Dawley rats weighing 200–300 g were housed under 12 h light/dark cycles at 23 °C and 60 ± 5% humidity. All animals had free access to food (Samyang Foods, Seoul, Korea) and water. Neuronal cells were isolated from neonatal rat brains (Day 1) in PBS (0.1 mol). Briefly, neuronal cells were removed and incubated for 15 min in Ca^2+^- and Mg^2+^-free Hanks’ balanced saline solution (Life Technologies) containing 0.2% trypsin. Cells were dissociated by trituration and plated into polyethyleneimine-coated plastic or glass-bottomed culture dishes containing minimum essential medium with Earle’s salts supplemented with 10% heat-inactivated fetal bovine serum, 2 mM l-glutamine, 1 mM pyruvate, 20 mM KCl, 10 mM sodium bicarbonate, and 1 mM HEPES (pH 7.2). Following cell attachment (3–6 h after plating), the culture medium was replaced with neurobasal medium containing B27 supplements (Life Technologies). The cells were cultured in neuronal cell culture medium for 3 days, and then further cultured in neuronal cell culture medium with or without 20% astrocyte culture media. Experiments were performed with 4- to 6-day-old cultures. More than 90% of the cells in these cultures were neurons, and the remainder were astrocytes as judged by cell morphology and immunostaining with antibodies against neurofilaments and glial fibrillary acidic protein (GFAP).

### 4.11. Western Blotting

Equal amounts of total protein (20 μg) were loaded and electrophoresed on a 10% SDS-polyacrylamide gel, and then transferred to a PVDF membrane (Hybond ECL, Amersham Pharmacia Biotech Inc., Piscataway, NJ, USA). After blocking in 5% (*w*/*v*) non-fat dried milk dissolved in Tris-buffered saline (10 mM Tris, 150 mM NaCl, and 0.05% tween-20; pH 7.8), the membrane was incubated with specific primary antibody against COX-2 (NB100-689, RRID:AB_10001091, 1:1000, 1 μg/mL, Novus Biologicals, Inc., Littleton), iNOS (NBP1-62139, RRID:AB_11018073, 1:1000, 1 μg/mL, Novus Biologicals, Inc., Littleton), Iba-1 (ab5076, RRID:AB_2224402, 1:1000, 1 μg/mL, Abcam, Inc., Cambridge, MA, USA), MAO-B (ab137778, 1:2000, 1 μg/mL, Abcam), TH (AB1542, RRID:AB_90755, 1:1000, 1 μg/mL, Millipore), GFAP (sc-33673, RRID:AB_627673, 1:1000, 200 ng/mL, Santa Cruz Biotechnology Inc., Santa Cruz, CA), p38 (sc-81621, RRID:AB_1127392, 1:1000, 200 ng/mL, Santa Cruz Biotechnology Inc., Santa Cruz, CA), phospho-p38 (sc-166182, RRID:AB_2141746, 1:1000, 200 ng/mL, Santa Cruz Biotechnology Inc.), JNK (sc-7345, RRID:AB_675864, 1:1000, 200 ng/mL, Santa Cruz Biotechnology Inc.), phospho-JNK (sc-6254, RRID:AB_628232, 1:1000, 200 ng/mL, Santa Cruz Biotechnology Inc.), and β-actin (sc-81178, RRID:AB_2223230, 1:1000, 200 ng/mL, Santa Cruz Biotechnology Inc.) for 2 h at room temperature. The blots were then incubated in the corresponding horseradish peroxidase-conjugated immunoglobulin G (Santa Cruz Biotechnology Inc.). The immunocomplex was detected by the ECL detection system. The relative density of the protein bands was quantified by densitometry using Electrophoresis Documentation and Analysis System 120 (Eastman Kodak Com., Rochester, NY, USA). The density of each band was normalized to background and β-actin.

### 4.12. Cell Viability Assay 

Primary cultured cells were plated in 96-well plates for 24 h, and were then treated with MMPP (1, 5, 10 μg/mL) for 24 h. After treatment, cell viability was evaluated using a WST-8 assay (Dojindo Laboratories, Tokyo, Japan). A yellow product (formazan) was formed through reaction with WST-8 [2-(2-methoxy-4-nitrophenyl)-3(4-nitro-phenyl)-5-(2,4-46953 disulfo-phenyl)-2H-tetrazolium monosodium salt] by dehydrogenases in the cell culture medium. The amount of dehydrogenase-induced formazan dye in cells was directly proportional to the number of living cells. In brief, 1 × 10^4^ cells per well were plated into 96-well plates, incubated at 37 °C for 24 h, and given fresh medium. Cells were then incubated with or without MPP^+^ (0.5 mM) in the absence or presence of various concentrations of MMPP at 37 °C for an additional 24 h. Next, 10 μL of the WST-8 solution was added to the wells, and incubation was continued for another 1 h. The resulting color was assayed at 450 nm using a microplate absorbance reader (SunriseTM, TECAN, Switzerland). 

### 4.13. MAO-B Activity Assay 

MAO-B activity was determined by monoamine oxidase activity using an Amplex^®^ Red Monoamine Oxidase Assay Kit (Molecular Probes, Cat. #A12214, Eugene, OR, USA). Briefly, the brain homogenates were incubated with benzylamine, a MAO-B substrate, with or without 1 μM selegiline for 30 min at 37 °C. The reaction was performed with equal volume of working solution at room temperature for 20 min. Fluorescence generated by H_2_O_2_ through 10-acetyl-3,7-dihydroxyphenoxazine in a horseradish peroxidase-coupled reaction was measured using a SpectraMax Gemini EM microplate spectrophotometer (Molecular Devices, LLC., SomJose, CA) with detection in the 530–560 nm range for excitation and 590 nm for emission.

### 4.14. Luciferase Assay 

Cultured cells were plated in 12-well plates (1 × 10^5^ cells/well) and transiently transfected with STAT3-Luc plasmid (Stratagene, La Jolla, CA), using a mixture of plasmid and Lipofectamine 3000 in OPTI-MEM according to the manufacturer’s specifications (Invitrogen, Carlsbad, CA, USA) for 24 h. The transfected cells were treated with MPP^+^ with or without MMPP for another 24 h. Luciferase activity was measured using a luciferase assay kit (Promega, Madison, USA) and a luminometer as described by the manufacturer’s specifications (WinGlow, Bad Wildbad, Germany).

### 4.15. Statistical Analysis

Due to the small sample size, we were not able to assume how well normality and equal variances fit. Sample size was not pre-determined by formal power analysis statistical methods. No samples or data were excluded from the analysis. The sample number for each experiment is stated in the figures and results. For the measurement of the image data, ImageJ (Wayne Rasband, National Institutes of Health, Bethesda, MD) was used. The data represent the mean ± SD. Statistical analysis of the data was carried out using analysis of variance [53] for repeated measures, followed by Dunnett’s post-hoc analysis using GraphPad Prism 5 software (Version 5.02, GraphPad software, Inc., La Jolla, CA, USA). Probabilities less than 5% (*p* < 0.05) were considered statistically significant.

## Abbreviations

MMPP	(E)-2-methoxy-4-(3-(4-methoxyphenyl) prop-1-en-1-yl) phenol
MPTP	1-methyl-4-phenyl-1,2,3,6-tetrahydropyridine
MPP+	1-methyl-4-phenylpyridinium
STAT3	signal transducer and activator of transcription 3
Iba1	including ionized calcium binding adaptor molecule 1
iNOS	inducible nitric oxide synthase
GFAP	glial fibrillary acidic protein
MAPK	mitogen-activated protein kinase
PD	Parkinson’s Disease
DA	Dopamine
AD	Alzheimer’s disease
ALS	amyotrophic lateral sclerosis
JNK	c-Jun N-terminal kinases
BHPB	(E)-2,4-bis(p-hydroxyphenyl)-2-butenal
DOPAC	3,4-Dihydroxyphenylacetic acid
MAO-B	monoamine oxidase B
WDI	World Drug Index
CMC	chemistry, manufacturing, and control
